# MobileNet-SVM: A Lightweight Deep Transfer Learning Model to Diagnose BCH Scans for IoMT-Based Imaging Sensors

**DOI:** 10.3390/s23020656

**Published:** 2023-01-06

**Authors:** Roseline Oluwaseun Ogundokun, Sanjay Misra, Akinyemi Omololu Akinrotimi, Hasan Ogul

**Affiliations:** 1 Department of Multimedia Engineering, Kaunas University of Technology, 44249 Kaunas, Lithuania; 2 Department of Computer Science, Landmark University, Omu Aran 251103, Kwara, Nigeria; 3 Department of Computer Science and Communication, Østfold University College, 1757 Halden, Norway; 4 Department of Mathematical Sciences, Augustine University Ilara-Epe, Lagos 106103, Lagos, Nigeria

**Keywords:** breast cancer histology, Internet of Medical Things, deep convolutional neural network

## Abstract

Many individuals worldwide pass away as a result of inadequate procedures for prompt illness identification and subsequent treatment. A valuable life can be saved or at least extended with the early identification of serious illnesses, such as various cancers and other life-threatening conditions. The development of the Internet of Medical Things (IoMT) has made it possible for healthcare technology to offer the general public efficient medical services and make a significant contribution to patients’ recoveries. By using IoMT to diagnose and examine BreakHis v1 400× breast cancer histology (BCH) scans, disorders may be quickly identified and appropriate treatment can be given to a patient. Imaging equipment having the capability of auto-analyzing acquired pictures can be used to achieve this. However, the majority of deep learning (DL)-based image classification approaches are of a large number of parameters and unsuitable for application in IoMT-centered imaging sensors. The goal of this study is to create a lightweight deep transfer learning (DTL) model suited for BCH scan examination and has a good level of accuracy. In this study, a lightweight DTL-based model “MobileNet-SVM”, which is the hybridization of MobileNet and Support Vector Machine (SVM), for auto-classifying BreakHis v1 400× BCH images is presented. When tested against a real dataset of BreakHis v1 400× BCH images, the suggested technique achieved a training accuracy of 100% on the training dataset. It also obtained an accuracy of 91% and an F1-score of 91.35 on the test dataset. Considering how complicated BCH scans are, the findings are encouraging. The MobileNet-SVM model is ideal for IoMT imaging equipment in addition to having a high degree of precision. According to the simulation findings, the suggested model requires a small computation speed and time.

## 1. Introduction

Throughout the world, one of the most important challenges relating to public health is cancer. With 12.5% of all new instances of cancer worldwide each year, breast cancer (BC) is currently the most prevalent cancer in the world. In the U.S., women are anticipated to receive diagnoses for 287,850 new instances of invasive BC and 51,400 new cases of non-invasive (in situ) BC in 2022. It is anticipated that 2710 new instances of invasive BC will have been identified in men. About 1 in 833 men may develop BC in their lifetimes. In the United States, there are more than 3.8 million women who have had breast cancer in the past. Women who are receiving treatment now and those who have concluded therapy are both included in this. In American women, BC is the most often diagnosed type of cancer. About 30% of women’s newly diagnosed cancers are predicted to be BCs in 2022 [1,2]. In particular, BC is the most widespread malignancy and the main reason for cancer-related deaths in women globally [3]. As a result, early detection of this condition is essential to stop its spread and lower the incidence of female morbidity. The likelihood of survival can be significantly increased by early diagnosis. Breast cancer (BC) is a diverse illness made up of several entities with unique biochemical, histological, and clinical characteristics [4]. The development of these cancerous breast cells causes an outbreak that may spread to nearby healthy tissues [4]. Radiology pictures, including those from mammography, ultrasound imaging, and magnetic resonance imaging (MRI), are utilized in the first clinical screening of the condition [5,6]. However, it is possible that these non-invasive imaging techniques will not be effective in identifying the malignant spots. To do this, a more thorough analysis of the malignancy in BC tissues is classically performed using the biopsy procedure. As part of the biopsy procedure, tissue samples are collected, firmly fixed on tiny glass slides, and stained to cause the nuclei and cytoplasm more noticeable [7]. The ultimate diagnosis of BC is subsequently made by pathologists after a microscopic examination of these slides [7].

The human interpretation of intricately detailed histopathology pictures is, however, a laborious, time-consuming operation that is potentially error prone. Additionally, the categorization of these pictures based on morphological criteria is rather subjective, which results in an average diagnostic concordance of 75% [8] among pathologists. The use of computer-assisted analysis [5,7,9] is, therefore, crucial in helping pathologists interpret the pictures from histology. Lowering the inter-pathologist differences in diagnostic choices, specifically, increases the analysis accuracy of BC [7]. However, the intra-class variance and inter-class evenness inside of the histopathological pictures of BC may not be adequately challenged by the standard electronic investigative methodologies, instigating from rule-based systems to ML approaches [10]. Additionally, the majority of these procedures count on the feature extraction techniques such as scale-invariant feature transform [10], rapidity robust features [11], and local binary patterns [12], all of which are founded on supervised data and are susceptible to producing biased outcomes when used to classify BCH images [13]. The demand for effective breast cancer disease analysis motivates the implementation of a sophisticated collection of computer models built on numerous layers of nonlinear processing units [14].

Delays in the discovery of illnesses can be brought on by improper medical examination, missed follow-up appointments, and trouble accessing a patient’s medical data [15]. The IoMT technology that has been used for a wide range of services is now being used in the healthcare industry. The way that humans and medical equipment interact when delivering medical services is undoubtedly changing under the IoMT. For patients, health specialists, clinics, pharmacological companies, and insurance organizations, IoMT-built healthcare applications are advantageous. Applications founded on the IoMT for health care are crucial because they enhance patient care while lowering facility costs [15]. 

The IoMT enables operative and efficient patient health monitoring, can even make disease detection early, and can even save a person’s life by starting therapy promptly [16]. The obtainability of modern sensors has enhanced the capabilities of IoMT facilities by making it possible to accurately and promptly gather a person’s physiological data. However, the diagnosis system’s accuracy depends on both the image analysis method and exact data in addition to both. DL-based approaches for job scheduling and sequencing for IoMT-centered health structures have also been created by researchers [17]. The procedures necessary for the conventional way of sickness diagnosis are quite expensive and time consuming. A skilled individual must collect the abnormal material with the aid of the computer-aided diagnosis system, which pathologists presently use to assist in monitoring and examining of patients’ health conditions [17]. The report written by the pathologist is founded on their evaluation of the examples, and practitioners refer to the reports. There are situations when a dearth of qualified pathologists might impede timely medical action and postpone the accurate diagnosis of illnesses, threatening the patient’s life. The need for automatic detection of medical pictures grows as a result [18]. 

The study of diseased pictures has gained significant attention as a result of the massive rise in AL and ML. With the increase in serious illness cases worldwide, there is a growing demand for effective computer-assisted disease detection. Given that the cellular structure in photographs differs in relation to color, forms, magnitudes, and other functional aspects, automated analysis of digital photos of a tissue sample presents several technical challenges. The accuracy of illness screening has increased thanks to the application of DL in computer-centered analysis. Disease detection using histological tissue pictures is now possible because of the development of extremely correct learning networks such as ConvNet/CNN and Recurrent Neural Network (RNN) [19].

Due to their capacity to inevitably learn complex and sophisticated patterns from images, DL approaches [20,21] have recently made extraordinary strides in computer vision, particularly in biomedical image processing. This has encouraged numerous investigators to use these approaches in the classification of BCH images. Due to the propensity to proficiently communicate parameters transversely through numerous layers of DL approaches, CNN in particular is generally exploited in image-associated tasks. However, AlexNet [22] is regarded as one of the foremost deep CNNs to attain notable accuracy on the ImageNet Large Scale Visual Recognition Challenge (ILSVRC) around 2012. Several CNN-built designs have been suggested all through the previous few ages. The notion of utilizing deeper networks with reduced convolutional filters were subsequently presented by VGG architecture [23], which won second place at ILSVRC 2014. The inception Network [24] and residual network (ResNet) [25] are two pre-trained models that take advantage of the notion that several weighted reduced convolutional filters might generate an effective receptive field.

Examining pathological pictures has placed a lot of emphasis on cytological image analysis for illness identification using computers. Unlike histopathological samples, which are more difficult to analyze, these pictures are typically characterized by lone or grouped cells. An extra thorough scan of the illness and its effects on tissue trials may be seen in the histopathological photos. Aside from that, screening histopathological pictures for illness analysis is regarded as the gold standard since it is effective at identifying a wide range of illnesses and tumors [26]. However, the difficulties of extra pathological features showing in the BCH scans are challenging to the automated disease detection procedure but can be handled using advanced AI-based approaches. These image analysis techniques can help doctors identify the actual cause of a patient’s illness and classify morphological traits associated with prognosis. The early and precise diagnosis of illnesses in IoMT applications might greatly benefit from automated image analysis; however, owing to the enormous size of such software, the processing is only feasible on cloud servers or fog nodes. 

IoMT devices can be equipped with small, lightweight software to calculate the pictures locally. A large number of the current research, as described in Section 2, have achieved excellent illness detection accuracy, but they are computationally costly, rendering them unsuitable for use in medical devices. To analyze histopathology samples, this research suggests a unique DTL MobileNet model with fewer parameter sizes and a significantly lower computing need. The final prognosis is produced by majority voting. Utilizing quantization, the model is further compacted without significantly affecting its performance.

In this study, we used two alternative lightweight DTL architectural techniques to effectively classify the histology of BC using data from the Kaggle breast cancer repository. The following is a summary of the paper’s key contributions:

- The study proposed a novel MobileNet-SVM model that combines MobileNet DTL and SVM ML model for BCH scans. This is the first of its kind to the best of our knowledge.
- A proposed DTL-based BCH image processing model learns critical characteristics from real-world BreakHis v1 400× BCH samples necessary for the disease’s detection.
- A new model is suggested with minimal size and computing power requirements so that it may be efficiently integrated into any medical image capture equipment and utilized to process the data correctly at the source.
- The models for the two lightweights were adjusted to increase the precision of training, validation and testing.
- The performance of the models was assessed as fully trained frameworks.
- When applied to a real-life health dataset, the implementation findings confirm the conclusions of the suggested MobileNet-SVM models. The comparison statement in the resulting unit demonstrates the model’s effectiveness in predicting illness using histopathological scans.

Therefore, the accurate classification of the breast malignant class on a precedence foundation is our major goal. We discovered that the combination of the MobileNet and SVM approaches, when fine-tuned, performed well in classifying benign and malignant BreakHis v1 400× BCH pictures.

The rest of this article is alienated into subsequent sections: related works are mentioned in Section 2, and summarizes the purpose of the study. The tools and models used to carry out this investigation are described in Section 3 of the paper. Section 4 discusses the investigational strategy, and confers the implementation outcomes and analyses. The discussion of the results in presented in Section 5. Section 6 concludes the investigation and suggests a course for the future.

## 2. Related Works

Medical cases can be studied using a variety of medical imagery processes, including ultrasonography (USG), computed tomography (CT), magnetic resonance imaging (MRI), and digitally scanned histological pictures. The field of deep learning-based medical image analysis has grown tremendously during the previous several decades. The study municipal has devoted itself to modeling AI-centered models for the detection of lethal illnesses, notably various types of cancer, to avoid patient fatalities owing to late diagnosis. When working with huge volumes of data, DL-based categorization approaches offer findings that are more practical, exact, and quick, as mentioned in [27,28]. Bayramoglu, Kannala, and Heikkila [29] suggested two independently sized CNN models for classifying histopathological BC pictures.

A cloud-integrated Android app for the detection of breast cancer from a BCH image was proposed by Chowdhury et al. [27] in 2022. For the prediction of BC, they utilized ResNet 101, a transfer learning-based model that was trained on 15,616 pictures and tested on 3904 images. The model’s precision and accuracy were tested on 3904 photos, and they were both 99.58%. A novel rank-based ensemble method was proposed by Majumdar, Pramanik, and Sarkar [28] in 2022 by merging the results of three transfer learning CNN models: GoogleNet, VGG11, and MobileNetV3 Small. To classify breast histopathology pictures into two categories, the suggested ensemble model is created utilizing the Gamma function. With classification accuracy scores of 99.16%, 98.24%, 98.67%, and 96.16% for 40×, 100×, 200×, and 400× levels of magnification, respectively, on the publicly available standard dataset known as BreakHis and 96.95% on another well-known dataset known as ICIAR-2018, our method outperforms state-of-the-art approaches.

The classification phase of pre-trained networks, which has not received enough attention, was the focus of Abbasniya et al. [30] study on sixteen different pre-trained networks. Among all studied convolutional neural networks, the Inception-ResNet-v2, which combines the benefits of residual and inception networks, has demonstrated the best feature extraction capabilities for BCH images (CNNs). The grouping of Categorical Boosting (CatBoost), Extreme Gradient Boosting (XGBoost), and Light Gradient Boosting Machine (LightGBM) have provided the best average accuracy during the classification phase. The suggested method, IRv2-CXL, is evaluated using the BCH Image Classification (BreakHis) dataset, with experimental findings showing that IRv2-CXL performs better than existing state-of-the-art methods. A unique hybrid AlexNet-gated recurrent unit (AlexNet-GRU) model for the detection and classification of lymph node (LN) BC was deployed by Ahmad et al. [31] in 2022. To classify LN cancer samples, the authors employed a well-known Kaggle (PCam) dataset. Three models are examined and compared in this study: the suggested AlexNet-GRU, CNN-LSTM, and convolutional neural network GRU (CNN-GRU). The experimental results showed that the proposed model performed significantly better than CNN-GRU and CNN-LSTM models in terms of accuracy, precision, sensitivity, and specificity (99.50%, 98.10%, 98.90%, and 97.50%), reducing pathologist errors that occur during the diagnosis process of incorrect classification. The effectiveness of the suggested model is examined by comparison with other contemporary ML/DL algorithms, which demonstrates that the proposed AlexNet-GRU model is computationally effective. The author offers the AlexNet-BC model, a novel framework for classifying breast pathologies, relating to that of research conducted by Liu et al. [32]. The ImageNet dataset is used to pre-train the model, and an enhanced dataset is used to fine-tune it. To punish overconfident low-entropy output distributions and make the predictions suitable for uniform distributions, we also developed an improved cross-entropy loss function. The suggested method is then verified through a series of comparative studies using datasets from BreaKHis, IDC, and UCSB. The experimental results demonstrate that, at various magnifications, the suggested method outperforms the state-of-the-art methods.

A single-task CNN model for predicting malignancy level and a multi-task CNN model for simultaneously predicting magnification and malignancy level are both included in the suggested model. The single-task CNN model’s average identification rate for identifying benign and malignant tissue is 83.25%, whereas the multi-task CNN model’s average recognition rate for identifying benign and malignant tissue is typically 82.13% and 80.10% for magnification level prediction. On the “Wisconsin BC” dataset [33], conducted a comparative examination of many machine learning (ML) techniques, including “Support Vector Machine” (SVM), “Naive Bayes” (NB), “Decision Tree” (C4.5), and “K-Nearest Neighbor” (K-NN). With an accuracy of 97.13%, SVM outperformed all other models. For the diagnosis of BC, Transfer Learning (TL) also showed promise. On the “BreakHis” dataset [34], utilized the TL idea utilizing AlexNet CNN architecture for classifying malignant and benign tumors using HI. With Google’s “Inception V3” for BC categorization [35], used augmentation and TL approaches to address the issue of restricted data availability. The accuracy of this model was 89%. Reference [36] proposed a DL framework for the TL technique-based identification and classification of cytology breast pictures. 

The features were extracted using CNN-based pre-trained models, including “GoogLeNet”, “VGGNet”, and “Residual Networks” (ResNet), which were then fed to the Fully Connected (FC) layer for the classification job using the average pooling method. With an accuracy of 97.25%, the suggested framework fared better than all existing models. To extract the important visual traits for the classification of BCH images, ref. [37] introduced a multi-network classification framework. For feature extraction, pre-trained multi-network Deep CNN (DCNN) models, such as “DenseNet-121”, “ResNet-50”, “multi-level Inception V3”, and “multilevel VGG-16”, have been employed. The retrieved features had a high degree of dimension, which increased the cost of calculation. Therefore, the dimensional reduction has been accomplished using the “Dual Network Orthogonal Low-Rank Learning” (DOLL) model. Using the Ensemble Support Vector Machine (E-SVM) classifier, the authors employed fused features and a voting mechanism for classification [37]. To classify BC Histopathology Images, a non-linear Class Structure-based Deep CNN (CSDNN) model was proposed. To limit the feature similarity between images belonging to various classes, some feature space limitations were created and incorporated with CSDCNN. The accuracy of this model was 93.2% at the patient level and 93.8% at the image level.

To expand the training dataset due to the lack of images, patches must be extracted from high-quality histopathology images. Reference [38] created a patch-based CNN classifier (PBC-CNN) for identifying BC high-intensity imaging. Determining the severity of BC requires the ability to detect mitosis. Reference [39] created a Deep Learning CNN-based system for the MITOS Histopathological Imaging dataset’s mitotic identification. The foreground cellular structure from HI has been extracted using the Cluster-based segmentation method K-means. For feature selection, the CNN model has been applied, and for feature reduction, PCA (Principal Component Analysis) and Linear Discriminant Analysis (LDA) have been utilized. The SVM classifier has also been used to differentiate between mitotic and non-mitotic cells. For this methodology, the accuracy result was 96.8%. Reference [40] utilized two pre-trained DCNN models, Inception-V3 and Inception-Resnet-V2, for BC detection using the BreakHis dataset. To address the uneven distribution of photos in BC subclasses, various data augmentation techniques were used.

Additionally, the scientists created a brand new unsupervised auto-encoder (AE) model to perform clustering analysis on histopathology images and to lower the dimensionality of features retrieved by the Inception-Resnet-V2 model. Since invasive cancer cells have poor contrast and a comparable look to non-invasive regions, segmenting the area of interest (ROI) on invasive cancer whole slide images (WSIs) is a difficult task. Authors of reference [41] proposed the concept of TL and the skipped connection-based U-Net Auto-encoder for the segmentation of WSIs for invasive BC. Reference [42] suggested an ensemble learning approach to enhance BC classification. For the classification job, the investigators employed gene-expression data and BCH. Gene expression is 1D data; thus, using the Convex Hull method [43] and the t-Distributed Stochastic Neighbor embedding technique, it was transformed into picture data. 

In their 2018 study, Bardou, Zhang, and Ahmad [44] examined two machine-learning methods for automatically classifying breast cancer histology images into benign and malignant tumors as well as into subclasses of each. The first method relies on the extraction of a set of manually created features that have been encoded using two coding models (bag of words and locality-constrained linear coding) and trained by support vector machines, while the second method relies on the creation of convolutional neural networks. To improve the convolutional neural network’s accuracy, we have also conducted experimental tests on “handcrafted features + convolutional neural network” and “convolutional neural network features + classifier” configurations. Convolutional neural networks beat the manually created feature-based classifier, according to the results, where we were able to reach accuracy levels of between 83.31% and 88.23% for multi-class classification and between 96.15% and 98.33% for binary classification.

The usefulness of Multiple Instance Learning (MIL) for computer-aided diagnosis of breast cancer patients, based on the interpretation of histopathological pictures, is investigated by Sudharshan et al. [45], who also suggests a weakly supervised learning framework. Without having to label every instance, multiple-instance learning involves grouping instances (pictures) into bags (patients). We contrast several cutting-edge MIL techniques, including the original ones (APR, Diverse Density, MI-SVM, and citation-kNN) and more contemporary ones, such as a non-parametric method and a deep learning-based approach (MIL-CNN). The BreaKHis dataset, which is available to the public and contains roughly 8000 microscopic biopsy pictures of benign and malignant breast tumors from 82 people, is used for the research.

A CAD system is presented by Anwar et al. [46] in 2020 to categorize BC as benign or malignant. The four stages of the suggested CAD technique are pre-processing of the image, feature extraction and fusion, feature reduction, and classification. The ResNet Deep Convolution Neural Network (DCNN)-extracted fusion features of wavelets packet decomposition (WPD) and histograms of the oriented gradient are the foundation of the CAD (HOG). Next, principal component analysis was used to decrease the feature data (PCA). Finally, various separate classifiers are trained using the reduced features. The results reveal that a 97.1% accuracy rate was attained.

Convolutional neural networks were proposed by Bayramoglu, Kannala, and Heikkila [29] in 2016 to classify breast cancer histopathology images regardless of their magnification (CNNs). We suggest two distinct architectures: a single-task CNN that predicts malignancy and a multi-task CNN that simultaneously predicts both malignancy and the degree of picture magnification. On the BreakHis dataset, evaluations and comparisons with earlier findings are made. According to experimental findings, the performance of the magnification-specific mode was improved by our magnification-independent CNN approach.

In order to automatically classify hematoxylin-eosin-stained breast cancer microscope images into normal tissue, benign lesion, in situ carcinoma, and invasive carcinoma using the gathered dataset, Hameed, Garcia-Zapirain, Aguirre, and Isaza-Ruget et al. [47] developed a deep learning strategy. The results showed that their proposed model performed better than the baseline AlexNet model and the cutting-edge VGG16, VGG19, Inception-v3, and Xception models, with an accuracy of 98%.

The bulk of the research studies presented in this part is built on intricate structures that demand a lot of computing. Simple models for illness prediction, such as SVM, CNNor Naive Bayes, perform substantially less accurately than approaches that utilize deep learning. The related studies reviewed are summarized in Table 1.

## 3. Material and Methods

The importance of locally computerized histologic image processing in IoMT technologies has been emphasized in the earlier sections. Thus, a computer vision analysis software approach advocates for a remedy with very minimal storage and computing requirements while maintaining an insignificant drop in accuracy likened to the most sophisticated ones for classifying images now available. A lightweight image-centered classifier with low resource needs and good accuracy makes up our suggested illness prediction model. There are four steps to the suggested solution. The preprocessing phase comes first. The normalization approach is used to preprocess the picture datasets at this point. Additionally, the image augmentation approach is used to enhance the dataset that is already accessible and to help balance the uneven datasets. The training and validation processes make up the second step. In this step, the preprocessed data are utilized to train the “MobileNet-SVM” model that we have suggested. The proposed model’s performance is tested in the third step, known as the testing phase when 20% of the dataset is put aside to assess its effectiveness. Following this, a confusion matrix is used to calculate the model’s performance metrics on the testing dataset. The prediction of illnesses is carried out in the fourth phase, which is the last stage. This aids in evaluating how well the suggested method detects BreakHis v1 400× BCH. In this part, we go over each of these methods concerning the suggested model.

### 3.1. Preprocessing Stage

The pre-processing step is applied to every input scan of the BreakHis v1 400× to produce recognition accuracy that is more consistent and has additional quality. A huge image dataset was needed for the CNN approach’s massively repeated learning to avoid the risk of over-fitting. The initial BreakHis v1 400× image dataset dimension includes 700 × 460. The dataset has been scaled down to 224 × 224, which will improve the computation time and prediction results.

All images were normalized to ImageNet standards. Then the image collection has been categorized into two which are Benign and Malignant and was uploaded to the system’s local drive. They were verified to be properly and accurately uploaded using Python code in the Jupyter Notebook environment. There are a total of 1819 scans in this BreakHis v1 400× dataset out of which 588 scans are from Benign patients and 1231 scans from Malignant patients as shown in Table 2. The imbalanced dataset was balanced using data augmentation techniques [40] as shown in Table 2.

The dataset was split in train, validation, and test set at a ratio of 80:10:10. A total of 10% of the whole balanced BreakHis v1 400× is first split, after which the validation 10% is split from the remaining dataset then the remaining is used for the model training. Table 3 shows the number of BreakHis v1 400× images input for the training, validation, and testing set before and after augmentation for the system implementation.

The dataset has an uneven distribution of scans among the two classifications. A dataset’s imbalance may result in bias in favor of the dominant class. The study introduced data augmentation techniques on the dataset to prevent problems with class imbalance. To prevent complete duplication of the images, each of the images is then randomly rotated, sheared, zoomed, and flipped. Data augmentation was also carried out to enhance the data size to make the suggested model more resilient to feature transformation. Several studies have looked into the importance of data augmentation in deep learning, as this technique frequently does not modify image classes and allows for a large amount of data and the creation of more generic models.

### 3.2. Image Augmentation

**Figure 1:**
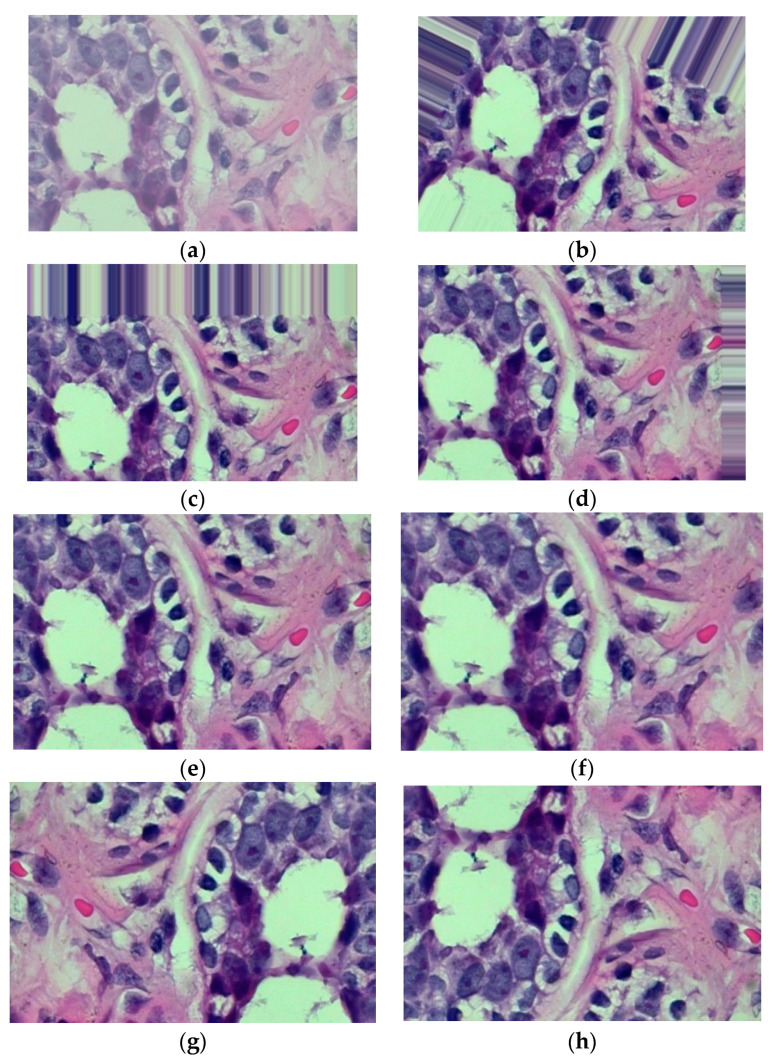
Various image augmentation methods for the same image (**a**). (**a**) Original image; (**b**) Rotated image; (**c**) Width shift range; (**d**) Height shift range; (**e**) Shear range; (**f**) Zoom range; (**g**) Horizontal Flip; (**h**) Vertical Flip.

By using significant modifications to the available data, image augmentation is a process often utilized to artificially upsurge the magnitude and diversity of the dataset. Additionally, it aids in resolving any datasets’ potential imbalanced data issues. Since datasets are the backbone of the suggested approach and imbalanced datasets might lead to devastating inaccuracies in our recommendations, having a broad and even dataset is crucial for image classification techniques. Images can be enhanced with data augmentation using a variety of techniques, including rotation, shearing, brightness shift, random zoom, horizontal flip, vertical flip, etc. However, it is still crucial to remember that not all data augmentation approaches can be used with all kinds of data. We ought to select image augmentation techniques to ensure that the modifications produce realistic images and maintain the caption of the source image. The authors chose to utilize horizontal flip, width shift range, height shift range, zoom range, and rotation range augmentations after carefully evaluating several image augmentation techniques, since these transitions will produce images that are probable to be found in an unidentified input image. The many augmentations utilized to produce the final image dataset for the investigation are depicted in Figure 1. To augment the image, the image was rotated at 45° (see Figure 1b), a width shift range of 0.2 (see Figure 1c), a height shift range of 0.2 (see Figure 1d), a shear range of 0.2 (see Figure 1e), a zoom range of 0.2 (see Figure 1f), a horizontal flip as True (see Figure 1g), or a vertical flip as True (see Figure 1h), as demonstrated in Table 4.

### 3.3. Proposed Model—MobileNet-SVM

Deep transfer learning is a sort of model that the authors have suggested, called MobileNet-SVM. The foundation of all cutting-edge image categorization technologies is the convolutional neural network (CNN). They are a form of neural network (NN) where convolution is used for at minimum a single layer instead of matrix multiplication. Contrary to the NN model, which treats each component of the source image as a standalone input, convolution takes neighborhood pixels into account, greatly enhancing the effectiveness of the network. MobileNets are built on a simplified design that creates lightweight DTL using depth-wise separable convolution layers. The foundation of the MobileNet framework is feature maps convolutions, which significantly impact a normal convolution into a depth-wise convolution (DWC) and an 11-convolution known as a pointwise convolution. The depth-wise convolution for MobileNets utilizes a single filter for every network interface. The outcomes of the DWC are then united by the pointwise convolution using an 11 convolution. Standard convolutions integrate inputs into a novel series of outputs in one iteration, while also filtering the inputs. This is divided into two layers by the depth-wise separable convolution, one for filtration and one for integration. This simplification results in a significant decrease in computational and framework size.

The same protocol was utilized for all three pre-trained CNN models, i.e., InceptionV3, MobileNet, and DenseNet121, implemented in this study at each stage of transfer learning. In this study, the four DTL models were selected based on a preliminary study carried out using previous investigations on the classification of breast cancer images, including InceptionV3, MobileNet, and DenseNet121. MobileNet was used out of the other lightweight model because we proposed an IoMT system and MobileNet was initially developed for a mobile device, which makes it process fewer number of parameters and lowers computational complexity.

**Figure 2:**
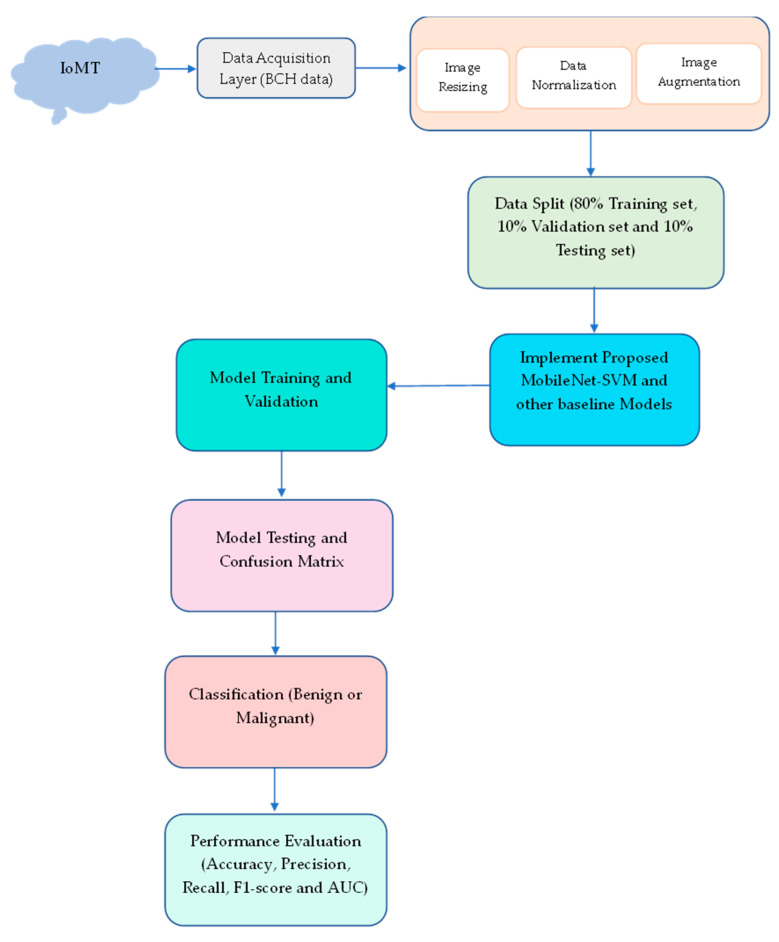
Proposed model flow diagram.

In this paper, a DTL is introduced for detecting BreakHis v1 400× BCH scans. To handle the issues of class imbalance in the BCH scan sample and provide variety, pre-processing and different image augmentation approaches are utilized in the foremost step. The BCH scans are classified into benign or malignant cancer in the second step after auto parameters are obtained and a pre-trained “MobileNet-SVM” model is put into use. Figure 2 displays the suggested technology’s process flow.

## 4. Implementation Results

This section explains the execution of the suggested model. It is divided into three subsections. In the first, the dataset which was used throughout the system execution is introduced together with innumerable problems with medical datasets and the way the study attempted to resolve them. The training, validation, and testing of the dataset on the MobileNet-SVM model are detailed in the second subsection. In the last subsection, the authors investigate and correspond the results of the proposed model with three existing baseline CNN architectures, which include DenseNet121, MobileNet, and InceptionV3. TensorFlow and Keras were used for the implementation process.

### 4.1. Dataset

**Figure 3:**
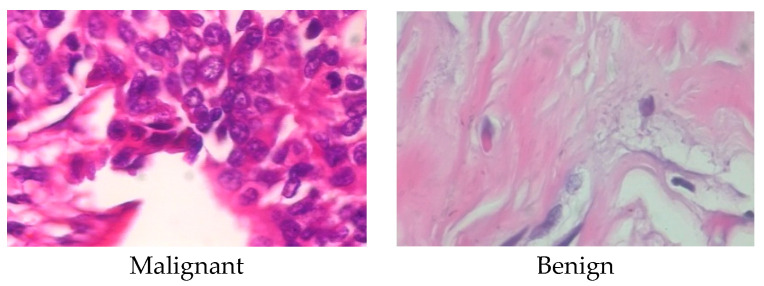
An example image of each class of BCH.

The BCH dataset [55] which the study has used consists of 588 scans of benign and 1231 scans of malignant samples, for a total of 1819 images of BCH. Figure 3 displays an example picture for each of the different subtypes. Techniques for image classifiers are trained on data that is very distinct from the accessible medical data. Medical datasets that are labeled are severely lacking. The ImageNet dataset, for instance, has more than 14 million photos in more than 20,000 distinct classifications. Similar medical databases, nevertheless, are hardly accessible to the common public. Additionally, the development of a comparable integrated medical dataset is difficult because the majority of medical conditions are distinct from one another and might call for a completely distinct method for their diagnosis.

Additionally, since the medical scans that are currently accessible typically have very high resolution, the common technique of image scaling to a smaller dimension is incorrect for them, since doing so could result in the loss of cellular features that are essential for the diagnosis of the illness. The dataset utilized in this study includes 375 high-resolution images. We used data augmentation to address these issues, primarily to increase the dataset size and address the issue of class imbalance. Table 5 displays the frequency of the BCH subclass of the dataset before and after the use of image augmentation approaches.

### 4.2. Transfer Learning

The process of transfer learning involves keeping the information learned when resolving an issue and using it to solve a new, related problem. When the dataset is small, this is particularly successful since it makes the model easier to react to the new data, which would not be as quick or effective if trained from scratch. In this study, the authors employ models with ImageNet pre-trained weights. The ImageNet is a collection of image data containing tens of millions of photos that are neatly organized and available to academics all over the world [56]. Additionally, the model’s fine-tuning was performed in this study. The fine-tuning step helps to achieve improvements in the model’s outcomes. It assumes that a model’s parameters must be altered very accurately for the model to respond to specific facts. A trained model or a portion of it is unfrozen during fine-tuning, and then training is carried out once more on the fresh data using a shallow learning rate. The weights that have already been learned are somewhat modified as a result. In this study, the encoder weights were frozen to allow for fine-tuning. According to [57], there are occasions when we can freeze the encoder and train only the de-coder that was randomly initialized to avoid damaging the weights that were properly trained with large gradients during the initial stages of training. Removing the last completely connected layer from selected pre-trained CNN models and replacing them with our new fully connected layer or layers is the primary method for fine-tuning (i.e., the same size as the number of classes in our new dataset). In this investigation, we employed two classes due to the benign B and malignant M instances in the imaging datasets.

### 4.3. Training, Validation, and Testing

There were three parts to the BreakHis v1 400× BCH dataset: training, validation, and testing. The validation and test sets were applied to assess the effectiveness of the proposed model, while the training set was applied to train the MobileNet-SVM method. As a result, the dataset was divided into the training, testing, and validation groups, with corresponding percentages of 80%, 10%, and 10%. The dataset described in Section 4.1 served as the basis for training the MobileNet-SVM. In total, 1993, 222, and 247 images, respectively, were utilized for training, validation, and testing on the BreakHis v1 400× BCH data. With training, validation, and testing accuracies of 100%, 92.34%, and 91.50% on the training and validation datasets, Table 6 displays the model parameter and values that produced the most efficient results. The study obtained a test accuracy of 91% on the test dataset using the implementation parameters (Table 6).

### 4.4. Performance Analysis

Following the training procedure, the computation method was evaluated using the test dataset. The accuracy, f1-score, precision, recall, and AUC-ROC curve values were employed to determine the effectiveness of the model. The evaluation metrics used in this study were investigated thoroughly as follows. TP represents true positives, TN for true negatives, FN for false negation, and FP for false positives in the concepts and formulas that are presented here.

#### 4.4.1. Accuracy (Acc.)

This measure calculates the proportion of accurate forecasts to all projections that were made correctly. The equation is shown in Equation (1):

(1) $$Accuracy=\frac{TP+TN}{\left(TP+TN+FP+FN\right)}$$

#### 4.4.2. Precision (Prec.)

Classification accuracy is not necessarily a reliable indicator of overall simulation results, as shown by several cases. One of these situations is when there is an uneven distribution of classes. It is absurd to achieve a superior accuracy rate if we regard all data as having the greatest caliber. Precision, on the contrary, implies that inconsistency may be obtained by using the exact tool again, such as when examining the same part. One such metric is precision, which is defined as:

(2) $$Precision=\frac{TP}{\left(TP+FP\right)}$$

#### 4.4.3. Recall

Another crucial metric is recalled, which is the division of the original dataset into classes that the algorithm accurately predicts. The recall is computed as:

(3) $$Recall=\frac{TP}{\left(TP+FN\right)}$$

#### 4.4.4. F1-Score

A popular indicator that combines recall and accuracy measurements is the F1- score. The F1- score is determined by:

(4) $$F1-score=\frac{2\times \left(Precision*Recall\right)}{\left(Precision+Recall\right)}$$

#### 4.4.5. AUC Score and ROC Curve

The receiver operating characteristic (ROC) is a probabilistic curve, and the area under curves (AUC) measures the degree of computational complexity. The relationship between specificity (rate of false positives) and sensitivity is shown in a graph by the ROC curve (true positive rate).

## 5. Discussion

The implementation with the suggested MobileNet-SVM model was executed on Python 3.9 using the Juypter Notebook interface. The Keras open-source library and the Tensor-flow platform were used to build the model. It employed a CategoricalCrossentropy loss function and the SGD optimizer with a baseline learning rate for training. The outcomes of the suggested MobileNet-SVM model focus on the following:

1. To discern amid benign and malignant dermoscopic scans.
2. To utilize a diversity of image augmentation procedures to determine the effectiveness of the given MobileNet-SVM on the BreakHis v1 400× BCH dataset.
3. To compare the outcomes of the proposed model with methods already in use.

### MobileNet-SVM Performance on BreakHis v1 400× Dataset

**Figure 4:**
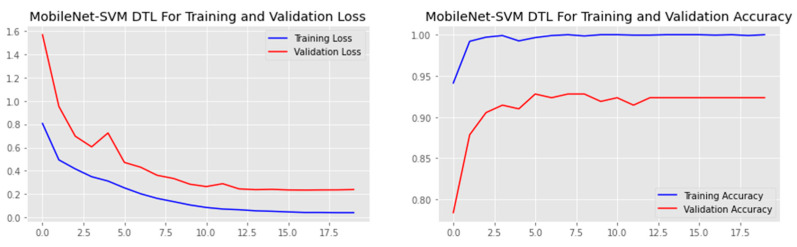
Accuracy and loss graph of the MobileNet-SVM model.

**Figure 5:**
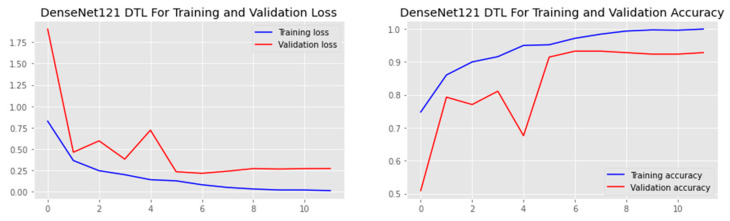
Accuracy and loss graph of the DenseNet121 model.

**Figure 6:**
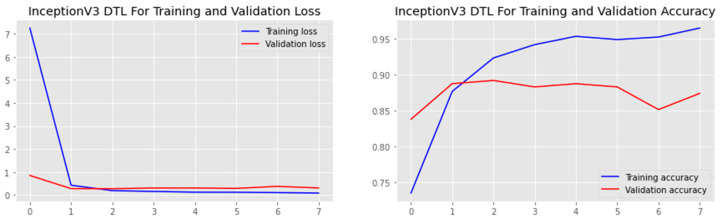
Accuracy and loss graph of the InceptionV3 model.

**Figure 7:**
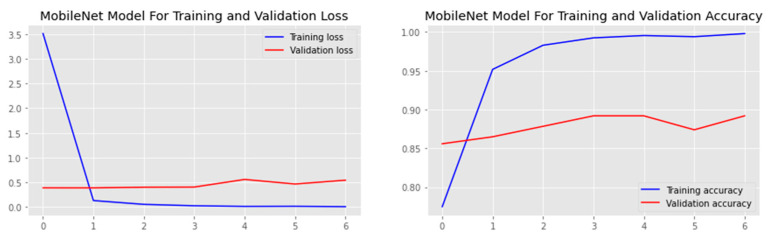
Accuracy and loss graph of the MobileNet model.

**Figure 8:**
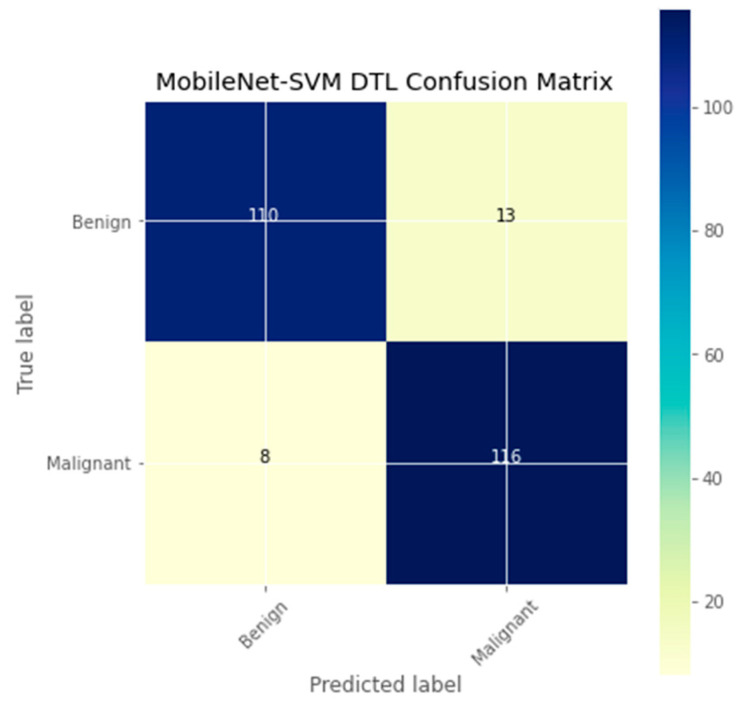
Confusion matrix of the MobileNet-SVM model.

**Figure 9:**
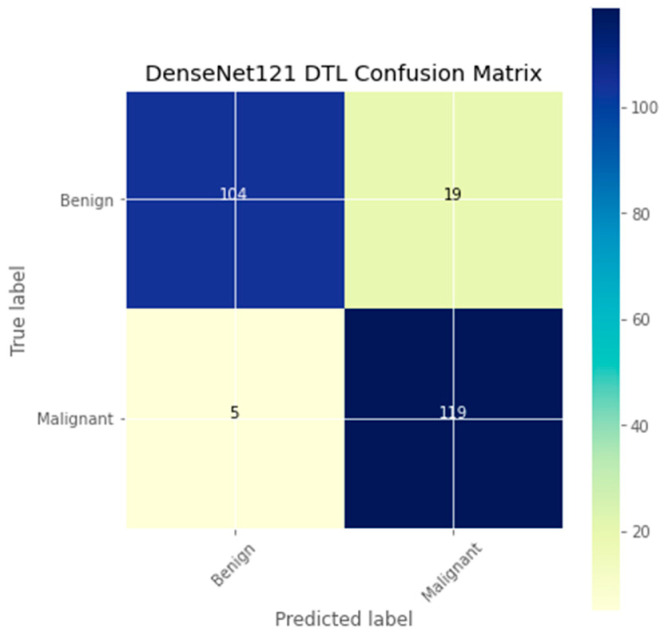
Confusion matrix of the DenseNet121 model.

**Figure 10:**
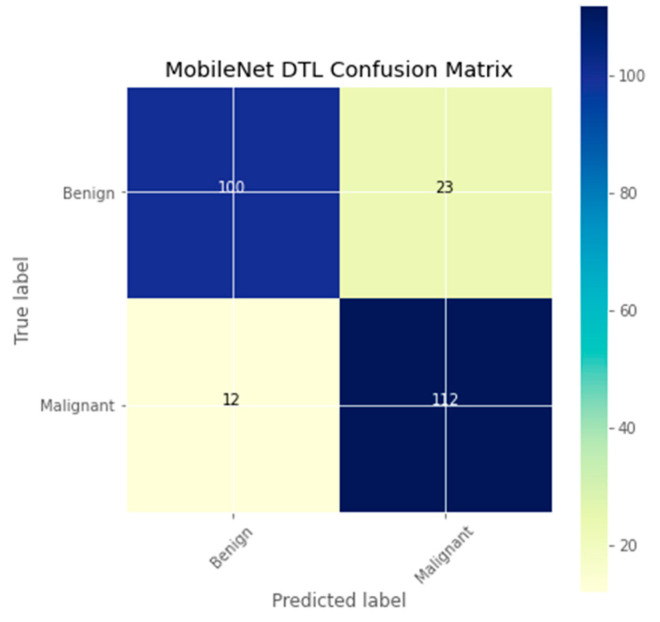
Confusion matrix of the MobileNet model.

**Figure 11:**
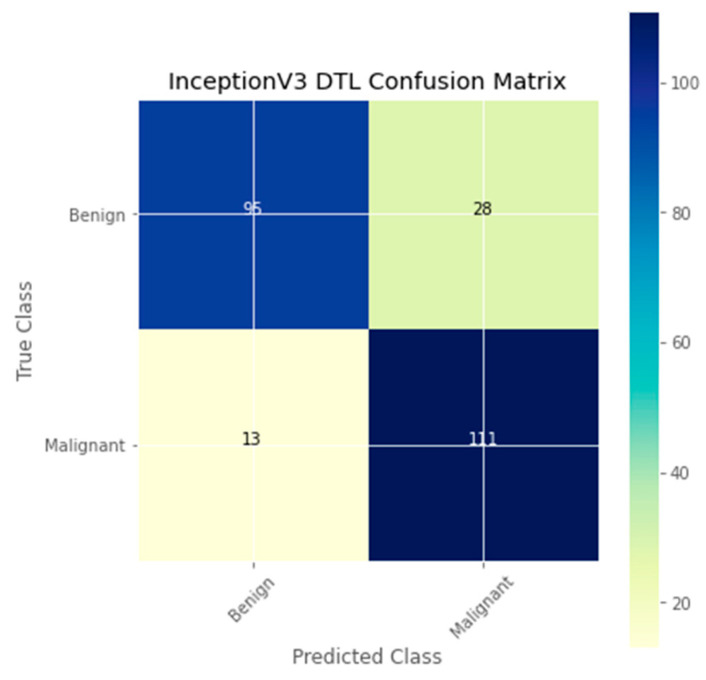
Confusion matrix of the InceptionV3 model.

**Figure 12:**
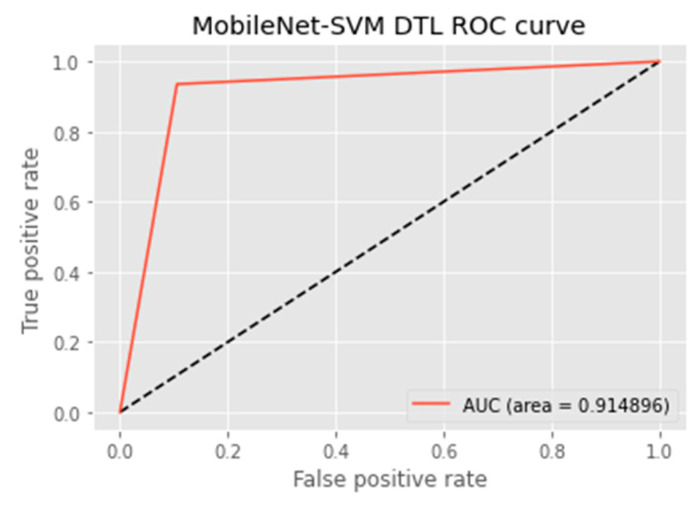
ROC curve of the MobileNet-SVM model.

**Figure 13:**
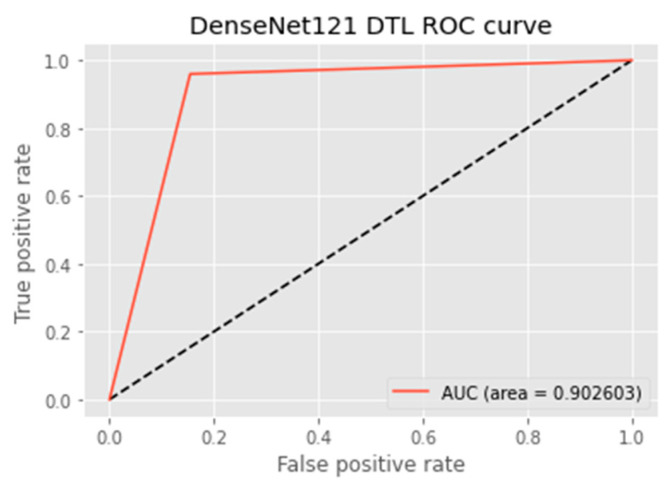
ROC curve of the DenseNet121 model.

**Figure 14:**
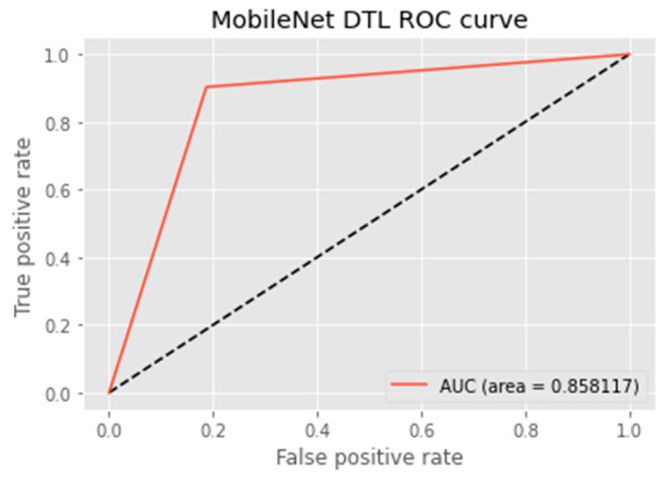
ROC curve of the MobileNet model.

**Figure 15:**
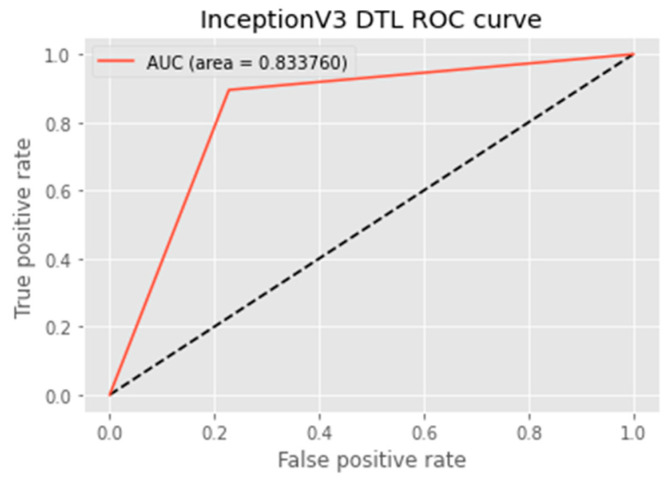
ROC curve of the InceptionV3 model.

The research aimed to appraise the effectiveness of the newly suggested MobileNet with SVM models. SGD optimizer, Categorical-Crossentropy loss function, 20 epochs, 32 batch size, and learning rate of 0.01 was used in the research, as depicted in Table 4. The accuracy and loss graphs for all the DTL approaches implemented are displayed in Figure 4, Figure 5, Figure 6 and Figure 7, while the confusion matrix of the approaches is shown in Figure 8, Figure 9, Figure 10 and Figure 11. Likewise, the AUC-ROC curves for all four DTL-implemented approaches are displayed in Figure 12, Figure 13, Figure 14 and Figure 15. The testing results showed that, for benign and malignant tumors, respectively, the new approach achieved 93% and 90% accuracy. On the BreakHis v1 400× dataset, it similarly attained an accuracy of 91.3%, as demonstrated in Table 3. On benign and malignant tumors, the transfer learning model had recall rates of 89% and 94%, respectively. It achieved an F1-score of 91% and 92% for benign and malignant tumors, respectively, with an accuracy of 89% and 94% for each. 

The confusion matrix is a useful machine-learning technique that assesses a model’s precision, recall, accuracy, and ROC curve. The categorization accuracy was measured graphically using a confusion matrix. Darker hue denoted the better classification accuracy of the MobileNet-SVM of the appropriate class, while lighter color denoted erroneously recognized data. In the confusion matrix, accurate estimates are shown diagonally, and inaccurate guesses are shown off-diagonally. The findings showed that the MobileNet-SVM model worked better when such a model was fine-tuned and used with the BCH dataset, as shown in Figure 8. It showed that out of 247 cancer scans, 116 were successfully diagnosed by the MobileNet-SVM model as cancerous images, while 110 were effectively labeled as benign images. The suggested MobileNet-SVM approach was generalized by the given MobileNet system, which had an accuracy result of 91.3% and a 1.0% error.

The MobileNet-SVM network displayed exceptional classification efficiency in the validation and test set classes attributable to a significant AUC (almost 91.5%). Using the ROC curve shown in Figure 12, the effectiveness of the suggested approach was evaluated. The ROC curve was denoted with blue. When the suggested technique is applied to the BreakHis v1 400× dataset with the data augmentation approaches and fine-tuning method applied in the training set, the evaluation metrics, including accuracy, F1-score, recall, precision, and the ROC curve, showed that the suggested model achieved remarkably well with an AUC of 0.9149.

Researchers evaluated the effectiveness of the suggested model with cutting-edge methods to reflect the scalability of the suggested methodology. As indicated in Table 7, it was established that the proposed hybrid DTL system surpassed baseline methods with an accuracy of 91.3%, precision of 89.4%, f1-score of 91.3%, AUC of 91.5%, and FPR value of 0.1008. However, the DenseNet121 baseline model performed best in terms of recall, i.e., of 95.4%. Upon evaluating the suggested model with cutting-edge techniques, there was little variance in misclassification. It can be deduced in Table 7 that the proposed model outperformed other baseline models because of the SVM that is combined with the MobileNet model. The proposed model also used regularization techniques, such as L1 and L2, for experimentation.

Table 8 shows the computational time complexity for the proposed models. It can be deduced that the baseline MobileNet had the lowest training time of 28 min, while the proposed model (MobileNet-SVM) had the second lowest computational time of 37 min. The time was increased 9 min because of the hybridization of the MobileNet model with the SVM technique. In general, it was deduced that the proposed model with 37 min still had the lowest computation time when compared with the other two baseline pre-trained model, with DenseNet121 having a training time of 62 min and InceptionV3 a training time of 49 min.

Table 9 compares the effectiveness of the suggested technique to various previously reported benign and malignant categorization systems. Compared to other recent studies, the study’s findings showed that the suggested approach had the best accuracy, as shown in Table 9. The suggested MobileNet-SVM approach classification results surpassed the prevailing research, such as studies by Singh et al. [58], obtaining 90.30% accuracy in classifying the BreakHis dataset using VGG-19 TL model. Spanhol et al. [59] had an accuracy of 86.1% using the AlexNet DL technique to classify BreakHis_400× images into benign and malignant diseases. Lastly, for the comparison process, Xie et al. [41] implemented Inception-ResNet-V2, and Inception-V3 DTL approaches on the BreakHis_400× dataset and obtained an accuracy of 84.50% and 82.08% respectively. Attallah et al. [52] used Histo-CADx model on BreakHis dataset to classify breast cancer and obtained an accuracy of 97.93% which means their system outperformed that of our proposed system. The results of the studies by Anwar et al. [46] and Choudhary et al. [60] outperformed our proposed system with an accuracy of 97.1% and 92.07%, respectively. Both studies were evaluated on BreakHis dataset using ResNet50 and ResNet with PCA models for their implementation, respectively. It was also discovered that the proposed study MobileNet-SVM outperformed two other existing studies that used BreakHis dataset. Sudharshan et al. [46] and Gupta and Bhavsar [61] used CNN and ResNet models respectively. The former obtained an accuracy of 88.03%, while the later had an accuracy of 88.25%.

Researchers, thus, claim that the suggested MobileNet-SVM model significantly outperforms the existing methods, by achieving a test accuracy of 91% on test dataset and 100% training accuracy using the training dataset. In comparison to other existing approaches, it acquired the best accuracy, as evidenced by Table 8. It is deduced from Table 8 that some studies performed better than the proposed model because their dataset was more robust than ours while the proposed model also outperformed some existing studies because of the introduction of data augmentation for balancing the dataset, regularization techniques, dropout technique to avoid model overfitting, and the introduction of the SVM model to combine with the MobileNet model. 

## 6. Conclusions

IoMT plays a critical role in delivering patients with high-quality, affordable, and rapid medical treatment. Early detection of serious disorders is also crucial to assisting this medical center. Millions of people’s lives can be saved if illnesses are detected earlier. However, an accurate and spontaneous illness diagnosis task has certain problems. Decisions may be delayed as a result of the transmission of real-time patient records to a subsequent IoMT level for computations. Additionally, the volume of histopathology pictures is often substantial, necessitating high bandwidth for data delivery. If the gathered data is examined remotely at the IoMT equipment, these problems can be overcome. We presented the MobileNet-SVM model, a high-performing, low-weight model that is equivalent to current existing approaches while preserving its compact size constraints, to address these problems.

BCH is among the deadliest types of cancer that affect women; however, if detected early enough, it may not be catastrophic. Therefore, it is crucial to use complementary imaging techniques that have been demonstrated to aid in diagnosis. These techniques are based on approaches created by medical professionals to find BCH before it expands to neighboring lymph nodes. In this study, the authors offer a MobileNet-SVM-based DTL model for benign and malignant BCH diagnostics that can be utilized to look into any possible disorders. The recommended approach is used to classify diseases as benign or malignant using photos of BCH disorders from the BreakHis v1 400× challenge dataset. The dataset was expanded using data augmentation methods, which also enhanced the precision of MobileNet-SVM. This system performs well and has a 91% percent diagnostic ability on the test dataset and 100% accuracy on the training dataset. Furthermore, the suggested model is used to compare the accuracy of several recently developed approaches. The proposed model was shown to offer exceptional prediction performance without the requirement for model retraining to increase model effectiveness. The study is limited to using only one benchmark dataset and is overfitted a little. It is, therefore, suggested that more than one BC dataset should be employed in the future and their performances can be evaluated to obtain the dataset that performs best. The problem of overfitting can be solved with the use of regularization techniques, batch normalization, dropout, early stopping techniques, learning rate, optimizer, more robust balanced dataset, and so on. Higher accuracies can be obtained with more training epochs, so it is suggested that the training epochs be increased in the future.

## Figures and Tables

**Table 1:** Summary of related works.

Authors	Models	Dataset	Accuracy	Limitations
Chowdhury et al. [27]	ResNet101	BreakHis dataset	99.58%	Used only one DL model for their study
Majumdar, Pramanik, and Sarkar [28]	GoogleNet, VGG11, and MobileNetV3	BreakHis dataset, ICIAR 2018	99.16%	Used only two benchmark datasets to evaluate their study
Ahmad et al. [31]	CNN-GRU	BreakHis dataset	99.50%	The study was evaluated against only two models
Liu et al. [32]	AlexNet-BC	BreakHis dataset	96.10%	The lesion area of the BCH images was not divided for the implementation of their study
Chang et al. [35]	Google’s “Inception V3	BreakHis dataset	89%	They employed only one DL model
Bardou, Zhang, and Ahmad [44]	SVM, Ensemble	BreakHis dataset	96.15% and 83.31	They limited their study to only two ML models
Sudharshan et al. [45]	APR, MI-SVM, KNN, MIL-CNN	BreakHis dataset	92.1%	They limited their works to one DL framework and they did not use any feature selection technique
Karthiga and Narasimhan [48]	K-means, Discrete Wavelet Transform, SVM	BreakHis dataset	93.3%	The accuracy achieved is less than 95%
Anwar et al. [46]	ResNet, PCA	BreakHis dataset	97.1%	Only one deep-learning algorithm was used
Senan et al. [49]	CNN, SVM and RF	BreakHis	99.67%, 89.84% and 90.55%	The proposed model was not compared and evaluated with existing systems.
Bayramoglu, Kannala, and Heikkila [29]	CNN	BreakHis dataset	83.25%	It is limited to only the baseline CNN
Aljuaid et al. [50]	ResNet 18, ShuffleNet and Inception-V3Net	BreakHis dataset	99.7%, 97.66%, and 96.94%	The datasets used in the study was not robust.
Liu et al. [51]	MSMV-PFENet	BreakHis dataset	93.0% and 94.8%	The study was not compared with existing systems
Attallah et al. [52]	Histo-CADx	BreakHis dataset	97.93	A multi-center study was not carried out to assess the performance of the proposed CADx system
Han et al. [53]	class structure-based deep convolutional neural network (CSDCNN)	BreakHis dataset	93.2	The study was not compared with other baseline DL techniques or existing studies.
Nahid and Kong [54]	CNN model containing residual block	BreakHis dataset	92.19%	The authors only used one type of breast cancer dataset

**Table 2:** Input BCH scan for the two classes.

Input BCH Scan	Before Image Augmentation	After Image Augmentation
Benign	588	1231
Malignant	1231	1231
Total	2462	2462

**Table 3:** Number of image dataset input for the training, validation, and testing sets.

Number of Input Images	Before Image Augmentation	After Image Augmentation
Training dataset	1473	1993
Validation	164	222
Testing dataset	182	247
Total	1819	2462

**Table 4:** Image augmentation approaches.

Changes	Settings
Rotation	45°
Width shift range	0.2
Height shift range	0.2
Shear range	0.2
Zoom range	0.2
Horizontal flip	True
Vertical flip	True

**Table 5:** Distribution of the images before and after image augmentation.

BCH Classes	Before Augmentation	After Augmentation
Benign	588	1231
Malignant	1231	1231

**Table 6:** Implementation parameters used.

Parameters	Values
Model used	MobileNet-SVM
Transfer form	From scratch transfer knowledge
Train layers	Layers 150–154
Optimizer	SGD
Learning rate	0.01
Activation function	Relu and softmax
Loss function	CategoricalCrossentropy
Batch size	32
Epochs	20

**Table 7:** Performance analysis of the proposed MobileNet-SVM on the BCH dataset.

DTL Model	Acc. (%)	Prec. (%)	Recall (%)	F1-Score (%)	AUC	FPR
**MobileNet-SVM**	**91.3**	**89.4**	93.2	**91.3**	**91.5**	**0.1008**
DenseNet121	90.3	84.6	**95.4**	89.7	90.3	0.1377
MobileNet	85.8	81.3	89.3	85.1	85.8	0.1071
InceptionV3	83.4	77.4	88.1	82.4	83.4	0.2014

**Table 8:** Computational time of the models.

Models	Time in Mins
MobileNet-SVM	37 min
DenseNet121	62 min
MobileNet	28 min
InceptionV3	49 min

**Table 9:** Comparative evaluation with existing models.

Authors	Models	Dataset	Accuracy (%)
Choudhary et al. [60]	ResNet50 Trasnfer Learning Model	BreakHis dataset	92.07
Singh et al. [58]	VGG-19 Transfer Learning Model	BreakHis dataset	90.30%
Spanhol et al. [59]	AlexNet	BreakHis dataset	86.10
Xie et al. [41]	Inception-ResNet-V2, Inception-V3	BreakHis dataset	84.50 and 82.08
Attallah et al. [52]	Histo-CADx	BreakHis dataset	97.93
Sudharshan et al. [46]	CNN	BreakHis dataset	88.03
Gupta, and Bhavsar [61]	ResNet	BreakHis dataset	88.25
Anwar et al. [46]	ResNet, PCA	BreakHis dataset	97.1

## Data Availability

The data are publicly available at https://www.kaggle.com/datasets/forderation/breakhis-400x (accessed on 15 October 2022).

## Abbreviations

BC	Breast Cancer
IoMT	Internet of Medical Things
IoT	Internet of Things
DTL	Deep Transfer Learning
BCH	Breast Cancer Histology
SVM	Support Vector Machine
ML	Machine Learning
CNN	Convolutional Neural Network
MRI	Magnetic Resonance Imaging
AUC	Area Under Curves
ROC	Receiver Operating Characteristics
